# Models of sequestration and receptor cross-talk for explaining multiple mutants in plant stem cell regulation

**DOI:** 10.1186/1752-0509-5-2

**Published:** 2011-01-05

**Authors:** Patrik Sahlin, Pontus Melke, Henrik Jönsson

**Affiliations:** 1 Computational Biology & Biological Physics, Lund University, Sölvegatan 14A, SE 223 62 Lund, Sweden

## Abstract

**Background:**

Stem cells reside in a plant's shoot meristem throughout its life and are main regulators of above-ground plant development. The stem cell maintenance depends on a feedback network between the *CLAVATA *and *WUSCHEL *genes. The CLAVATA3 peptide binds to the CLAVATA1 receptor leading to WUSCHEL inhibition. WUSCHEL, on the other hand, activates CLAVATA3 expression. Recent experiments suggest a second pathway where CLAVATA3 inhibits WUSCHEL via the CORYNE receptor pathway. An interesting question, central for understanding the receptor signaling, is why the *clavata1-11 *null mutant has a weaker phenotype compared with the *clavata1-1 *non-null mutant. It has been suggested that this relies on interference from the mutated CLAVATA1 acting on the CORYNE pathway.

**Results:**

We present two models for the CLAVATA-WUSCHEL feedback network including two receptor pathways for WUSCHEL repression and differing only by the hypothesized mechanisms for the *clavata1-1 *non-null mutant. The first model is an implementation of the previously suggested interference mechanism. The other model assumes an unaltered binding between CLAVATA3 and the mutated CLAVATA1 but with a loss of propagated signal into the cell. We optimize the models using data from wild type and four single receptor mutant experiments and use data from two receptor double mutant experiments in a validation step. Both models are able to explain all seven phenotypes and in addition qualitatively predict CLAVATA3 perturbations. The two models for the *clavata1-1 *mutant differ in the direct mechanism of the mutant, but they also predict other differences in the dynamics of the stem cell regulating network. We show that the interference hypothesis leads to an abundance of receptors, while the loss-of-signal hypothesis leads to sequestration of CLAVATA3 and relies on degradation or internalization of the bound CLAVATA1 receptor.

**Conclusions:**

Using computational modeling, we show that an interference hypothesis and a more parsimonious loss-of-signal hypothesis for a *clavata1 *non-null mutant both lead to behaviors predicting wild type and six receptor mutant experiments. Although the two models have identical implementations of the unperturbed feedback network for stem cell regulation, we can point out model-predicted differences that may be resolved in future experiments.

## Background

The development of animals and plants is dependent on undifferentiated stem cells residing in special locations called niches [1]. In a plant, stem cells are maintained in the shoot apical meristem (SAM) throughout its life, and the SAM is the source of all aerial parts of the plant [2,3].

Spatial regions of different expression patterns and functions are found within the SAM. The central zone is located at the tip of the apex and consists of slowly dividing stem cells expressing the *CLAVATA3 *(*CLV3*) gene. Due to cellular growth and proliferation, stem cells from the central zone move into the surrounding tissue where the spatial location of each daughter cell is a main determinant of cell fate. Located below the central zone is a small group of cells that are believed to form a control zone for the organization of the SAM. These cells express the *WUSHEL *(*WUS*) gene, which encodes a homeodomain transcription factor and has been shown to be required for maintaining stem cells in the shoot [4]. Although not expressed in the same cells, WUS and CLV3 regulate the expression of each other. While WUS upregulates CLV3, the intercellular peptide CLV3 acts together with the receptor kinase CLAVATA1 (CLV1) in a signaling pathway that downregulates WUS [4-7]. This feedback network is a main regulator of stem cell maintenance in the SAM. If the number of stem cells is low the CLV3 signal will be weak and expression of WUS will in-crease, which in turn will induce CLV3 expression. If instead there is an abundance of stem cells, WUS will be downregulated and CLV3 expression will decrease.

Additional molecules have been identified to be important for SAM development. The receptor-like CLAVATA2 (CLV2) is involved in WUS repression acting within the CLV3 signaling path-way [8]. Müller et al. (2008, 2009) [9,10] recently identified a kinase CORYNE (CRN) that interacts with CLV2 and together form a receptor for CLV3. In addition, the BAM family of receptors has an antagonistic effect compared to CLAVATA1 in stem cell regulation [11,12], and several members of the CLE (CLAVATA3/ESR-related) ligand family were shown to affect the SAM development in perturbation assays [13]. The intracellular components of the WUS-repressing signal are to a large extent unknown, but POLTERGEIST (POL) and POLTERGEIST-LIKE1 (PLL1) have been shown to be important for mediating the signal [14]. In addition, hormonal signaling and chromatin remodeling has been implicated in the regulation of WUSCHEL [15-17].

Reduction in CLAVATA signaling leads to increased WUS and CLV3 expressions and an increase in number of stem cells and shoot size [18] as well as an increased number of carpels produced in flowers. A number of *clv1 *alleles have been shown to have different strengths in these phenotypic traits. Somewhat unintuitively, the *clv1-11 *null-mutant was shown to have a weaker phenotype than the non-null *clv1-1 *mutant [19]. Müller et al. (2008) [9] also found that the *crn-1 clv1-1 *double mutant showed weaker phenotype than the *crn-1 clv1-11 *double mutant. It was suggested that the stronger phenotype of the *clv1-1 *mutant compared to the null mutant is due to a functional overlap between multiple receptors, and that the dominant effect of *clv1-1 *could relate to cross-talk with other receptors [19]. With the identification of CRN/CLV2, this receptor was suggested to be the target for interference by the mutated CLV1 [9].

Several theoretical models have been used to investigate different aspects of the stem cell regulatory network in the SAM [20]. For example, spatial models using static cell-based SAM templates have been used to investigate how the WUS-activated CLV3 expression region could be localized to the central zone, and how WUS may be spatially activated via a pattern-forming mechanism [21-23]. The intracellular WUS activation network has been further investigated showing the importance of the hormone cytokinin for WUS activation [15], and a cell-population based model has been used to investigate uncoupling of the sizes of the *CLV3 *and *WUS *domains at different growth conditions [24].

None of the published models have included multiple receptor pathways for the WUS-repressing signal, and in this paper we use computational modeling to investigate details of receptor and ligand turnover, interactions, and signaling. We focus on the differences in receptor mutants, where the main question is how a *clv1-11 *null mutant can have a weaker phenotype than a *clv1-1 *non-null mutant in the context of phenotypes for a number of single and double receptor mutants. We develop two models capturing the main aspects of the negative feedback loop for stem cell regulation. In the models, CLV3 binds to both CLV1 and CRN/CLV2 receptors, the bound receptors propagate a combined signal repressing WUS, and WUS induces CLV3 production. The two models only differ in the implementation of the *clv1-1 *non-null mutant. The first model is used to test the proposed idea of receptor interference as an explanation of the differences in phenotypes of *clv1 *mutants and the second model is used to test a more parsimonious loss-of-signal mutant hypothesis. A motivation for the second model is that the *clv1-1 *has been identified as a missense mutation in the kinase part of the receptor [19].

A common problem for modeling biological systems is the abundance of unknown values for the kinetic reaction parameters. To address this problem we use a parameter ensemble approach, where we for each model extract a number of parameter sets, chosen for the ability of the model (simulated with such a set of parameter values) to explain data from multiple mutant experiments [25]. For each model of the *clv1-1 *mutant, the parameter sets provide a semiglobal description of the model behavior, instead of a more parameter value dependent description that results if a single parameter set would be used.

We apply statistical tools on the parameter sets to obtain predictions about biological properties of the stem cell regulating network resulting from introducing the hypothesized mechanisms for the *clv1-1 *mutant.

## Results and Discussion

**Figure 1:**
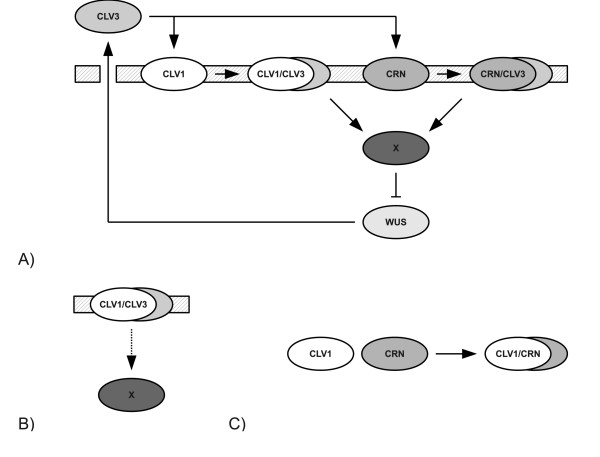
**Illustation of the stem cell regulation models**. A) A schematic drawing of the unperturbed stem cell regulation model. B-C) Schematic drawings of the two different *clv1-1 *mutant models. B) In the loss-of-signal model the strength of the signal from the active CLV1-receptor is affected. C) In the interference model the two receptors form a complex without function.

We implemented the stem cell regulatory network as a system of ordinary differential equations (ODEs) using standard mass action kinetics for molecular reactions (Figure 1, Methods). The model consists of two receptors, CLV1 and CRN (representing the CRN/CLV2 receptor), that when bound to CLV3 repress WUS expression, and WUS-induced CLV3 expression.

### Models of two clv1-1 hypotheses are both able to explain data from wild type and six receptor mutant experiments

To objectively obtain parameter values we compared the models with experimental data from wild type and the four receptor loss-of-function mutants *clv1-1*, *clv1-11*, *crn-1*, and *crn-1 clv2-1 *(Methods). We implemented *clv1-11 *and *crn-1 clv2-1 *as null mutants, removing the receptors from the model, and the *crn-1 *as a complete loss-of-signal mutant [9] (Methods). The *clv1-1 *mutant is implemented either as a loss-of-signal mutant (*loss-of-signal model*, Figure 1B) or by adding an interference mechanism acting on the CRN receptor pathway (*interference model*, Figure 1C). To compare phenotypic strength between a model and experiments, we used WUS levels as a measure in the models and compared with carpel numbers, which represent an experimental estimate of phenotypic strength, see e.g. [9,15,19] (Methods).

**Figure 2:**
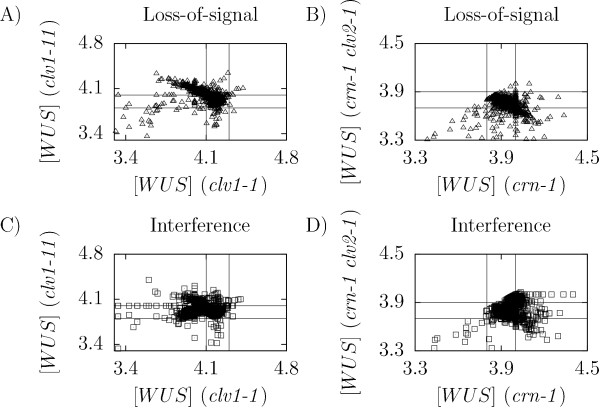
**WUS equilibrium expressions in simulations of the *clv1-1*, *clv1-11*, *crn-1*, and *crn-1 clv2-1 *single receptor mutants with the loss-of-signal (A-B) and the interference (C-D) models**. Parameter sets obtained from the optimization algorithm. Solid lines mark the regions that were selected in the optimization step (Table 1).

We used an optimization algorithm to find candidates for parameter values [26] and performed multiple optimizations to get an ensemble of parameter (value) sets for each of the two models (Methods). We performed 25,000 optimizations for each model and kept only parameter sets for which the model was able to reproduce data observed in the experiments (Figure 2). For the two models the optimization algorithm found 21,968 (loss-of-signal) and 23,121 (interference) valid parameter sets (Figure 2).

**Figure 3:**
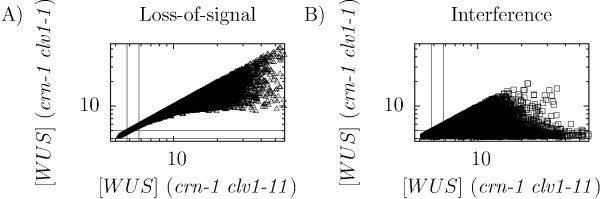
**WUS equilibrium expressions in simulations of the *crn-1 clv1-11 *and *crn-1 clv1-1 *double mutants with the loss-of-signal (A) and the interference (B) models with the parameter sets obtained from the optimization step**. Solid lines mark the regions that were selected in the validation step (Table 1).

Given that the number of parameters in the model exceeded the number of available experiments, it was important to lower the tendency of over fitting. Hence we applied double mutants as an a *posteriori *validation step, where we compared data from simulations with data from the two double loss-of-function and null-mutants *crn-1 clv1-11 *and *crn-1 clv1-1 *(Table 1), keeping only parameter sets for which the models were able to explain the double mutants (Figure 3). We observed that for most parameter sets the models showed too strong phenotypes for the double mutants (Figure 3), and after the validation only 118 (loss-of-signal) and 531 (interference) parameter sets remained. The strong double mutant phenotypes in the simulations can be explained by the lack of other dynamic input to the WUS repression in the model, except for the two pathways that are knocked out.

**Table 1:** Mutants

Mutants	Carpels/Flower	SE	Threshold error
Wild type	2.0	0.0	-
*crn-1*	3.9	0.1	0.1
*clv1-11*	3.9	0.1	0.1
*crn-1 clv2-1*	3.8	0.1	0.1
*clv1-1*	4.2	0.1	0.1

*crn-1 clv1-11*	5.3	0.1	0.5
*crn-1 clv1-1*	4.5	0.1	0.5

Experiments used in the optimization and validation steps to find parameter values. The first four mutants (above the line) are single-receptor mutants and are used in the optimization step. The remaining two double mutants are left for the validation step. Carpel number values and standard errors are from [9]. The threshold errors are the errors used to find valid parameter sets in the optimization and validation steps.

In conclusion, the loss-of-signal and interference models are both able to reproduce data seen in wild type, four single receptor, and two double receptor mutant experiments.

### The loss-of-signal hypothesis implies sequestration of CLV3 for the clv1-1 mutant

The difference between the two models is in the implementation of the *clv1-1 *mutant, which in each case is described by a single parameter - *k*_3, weak _for the loss-of-signal model and *k*_8 _for the interference model. For the loss-of-signal model, we observed that the average CLV1/CLV3 signal in the mutant (*k*_3_, weak) is an order of magnitude smaller than the wild type signal (*k*_3_) (Table 2), meaning that the model indeed shows a loss-of-signal behavior. Likewise, we observed for the interference model that the average interference strength in the mutant (*k*_8_) is an order of magnitude larger than the receptor turnover rates (*t*_1 _and *t*_2_) (Table 2). This simple sanity check confirmed that the hypothesized *clv1-1 *- mechanisms were effective in the models when using the parameter sets obtained from the optimization and validation steps.

**Table 2:** Model parameters

	Loss-of-signal		Interference	
Parameter	Mean value	Sensitivity	Mean value	Sensitivity
*k*_1_	2.6 ± 2.4	-0.35 ± 0.14	1.2 ± 0.6	-0.48 ± 0.10
*k*_2_	1.4 ± 1.0	0.12 ± 0.08	2.2 ± 1.4	0.32 ± 0.12
*k*_3_	3.6 ± 1.5	-1.0 ± 0.10	1.7 ± 0.9	-0.85 ± 0.13
*k*_4_	1.3 ± 0.9	-0.44 ± 0.10	1.0 ± 0.5	-0.49 ± 0.09
*k*_5_	2.3 ± 1.4	0.33 ± 0.12	2.3 ± 1.4	0.35 ± 0.10
*k*_6_	2.3 ± 1.2	-0.81 ± 0.08	1.8 ± 0.9	-0.77 ± 0.06
*k*_7_	1.2 ± 0.7	0.85 ± 0.13	1.3 ± 0.8	0.91 ± 0.09
*K*	2.47 ± 1.17	2.5 ± 0.6	2.2 ± 0.9	2.5 ± 0.7
*n*	5.1 ± 1.3	-0.19 ± 0.04	4.4 ± 1.2	-0.29 ± 0.08
*t*_1_	2.6 ± 1.2	0.61 ± 0.17	0.95 ± 0.48	0.32 ± 0.20
*s*_1_	1.3 ± 0.7	-0.62 ± 0.11	2.3 ± 1.0	-0.69 ± 0.08
*t*_2_	0.77 ± 0.55	0.24 ± 0.12	0.71 ± 0.36	0.21 ± 0.09
*s*_2_	1.9 ± 0.9	-0.67 ± 0.09	2.0 ± 0.9	-0.69 ± 0.05
*t*_3_	1.8 ± 0.9	-0.36 ± 0.21	3.9 ± 2.2	-0.15 ± 0.18
*s*_3_	2.1 ± 1.2	-1.2 ± 0.3	1.5 ± 0.6	-1.1 ± 0.2
*t*_4_	1.6 ± 0.8	1.8 ± 0.1	1.2 ± 0.6	1.6 ± 0.2
*s*_4_	0.65 ± 0.33	-0.76 ± 0.51	0.76 ± 0.35	-0.87 ± 0.59
*d_W_*	2.4 ± 2.2	-0.85 ± 0.12	2.1 ± 1.4	-0.90 ± 0.08
*k_W_*	1.4 ± 1.0	-0.15 ± 0.13	1.3 ± 0.84	-0.088 ± 0.088
*k*_3, weak_	0.15 ± 0.13	--	--	--
*k*_8_	--	--	5.6 ± 4.8	--

Model parameter properties for parameter sets remaining after the validation step. Mean value columns: Averages and standard deviations of parameter values. Sensitivity column: Averages and standard deviations of wild type WUS sensitivities (Eq. 21). (loss-of-signal 118 parameter sets, interference 531 parameter sets).

The stronger phenotype of the *clv1-1 *mutant requires that the CRN pathway signal is weaker in the *clv1-1 *mutant compared with the *clv1-11 *null mutant. For the interference model this requirement is fulfilled by the interference mechanism itself since CRN receptors are made unavailable for binding when interfered by the mutated CLV1 in the *clv1-1 *mutant.

**Figure 4:**
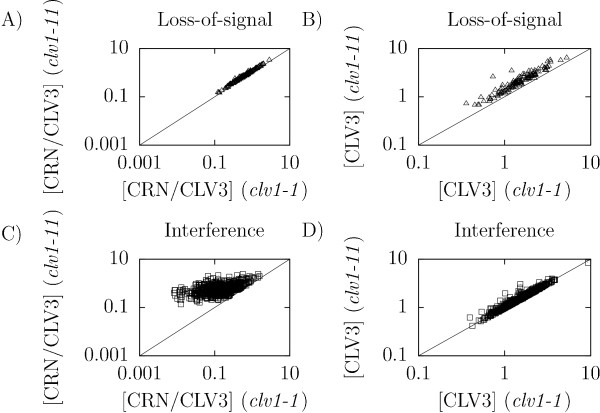
**Equilibrium concentrations of bound CRN receptors and free CLV3 in simulations of the *clv1-1 *and *clv1-11 *mutants of the two models**. A-B) Loss-of-signal model. C-D) Interference model. A) and C) Concentrations of bound CRN. B) and D) Concentrations of bound CLV3.

For the loss-of-signal mutant it is not as obvious how the CRN pathway signal can be weaker in the *clv1-1 *mutant than in the *clv1-11 *null mutant, but since the *clv1-1 *protein product still binds CLV3 it could sequester CLV3, making it unavailable for the CRN pathway. This would indirectly affect the signal strength of the CRN pathway. For the *clv1-11 *null mutant no CLV1 is present to bind to CLV3 leading to an increase of bound CRN receptors. To verify this intuitive explanation, we investigated the amounts of free CLV3 and bound CRN receptors in the different *clv1 *mutants. As expected we observed that both models had a lower amount of bound CRN receptors in the *clv1-1 *mutant than in the *clv1-11 *null mutant (Figure 4A and 4C), although the mechanisms for achieving this differed. Furthermore, the *clv1-1 *loss-of-signal mutant had lower levels of free CLV3, confirming the sequestration of CLV3 (Figure 4B). In contrast, for the interference model we observed no signs of CLV3 sequestration (Figure 4D).

Taken together these results show that the loss-of-signal model utilizes sequestration of CLV3 in the *clv1-1 *mutant in order to generate a stronger phenotype than the *clv1-11 *null mutant. The interference model, on the other hand, uses interference between the receptor pathways and simulations suggest that the model is less constrained by experimental data to achieve this result (cf. Figures 4A and 4C), as was also indicated by the number of parameter sets that passed the validation step for the two models.

### The two clv1-1 hypotheses leads to differences in properties of the unperturbed stem cell regulating network

Each model had a large ensemble of parameters after the optimization and validation steps. Since the wild type stem cell regulating networks of the two models are identical, there are no a *priori *reasons for the parameter values of the models - excluding the parameters unique for each implementation of the *clv1-1 *mutant - to differ. To analyze differences in parameter values we generated a hypothesis-neutral background distribution by optimizing the model without using the *clv1-1 *mutant. We performed 25,000 optimizations and for 24,686 parameter sets the model was able to reproduce the wild type and the three single mutant experiments. These parameter sets were used as a background parameter distribution for the two models in the proceeding analysis.

**Figure 5:**
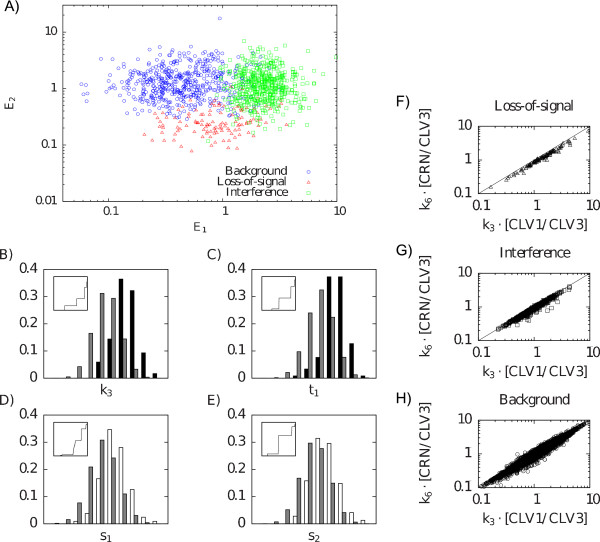
**Comparisons of parameter values between the loss-of-function and interference models and strengths between the CLV1 and CRN receptor pathways**. A) Parameter sets for the two models and the background distribution presented with the help of a PCA-analysis. The parameter sets are projected on the two largest principal components (*E*_1 _and *E*_2_). B-E) Distributions of parameter values of parameters highlighted in the analysis step. B-C) Comparing parameter value distributions of parameters *k*_3 _(B) and *t*_1 _(C) for the loss-of-signal model (black) and the background distribution (gray). D-E) Comparing parameter value distributions of parameters *s*_1 _(D) and *s*_2 _(E) for the interference model (white) and the background distribution (gray). Axes have log-scale and have ranges [0.23, 12] (B), [0.082, 9.9] (C), [0.24, 9.5] (D), and [0.21, 7.6] (E). Corresponding ROC-curves are presented in each plot with axes ranging from 0 to 1.0. F-H) The parameters *k*_3 _and *k*_6 _set the strengths of the signals from bound CLV1 and CRN receptors. To compare the strengths of the two receptor pathways we plot each parameter multiplied with respective concentration of bound receptors. The comparison is done for the loss-of-signal model (F), the interference model (G), and the background distribution (H). Number of parameter sets for which the CRN pathway is stronger than the CLV1 pathway: Loss-of-signal - 0 (0%), Interference - 17 (3.2%), Background - 1696 (6.9%).

We performed a *Principal Component Analysis *(PCA) on the joint parameters of the models. Interestingly, we observed that there was a clear separation both between the distributions coming from the two models, and between those of the individual models and the background distribution (Figure 5A). This showed that the introduction of the two hypotheses for the *clv1-1 *mutant leads to differences in the properties of the unperturbed stem cell regulating network.

To further analyze the differences between the models we compared each parameter individually between the two models and the background by using *receiver operating characteristics *(ROC) curves, where we used the area under the ROC-curve (AUC) to quantify differences between the three parameter distributions (Methods) [27]. We generated sorted lists of the AUC values (Table 3), and some parameters showed differences between either *clv1-1 *model and the background.

**Table 3:** AUC comparisons

Loss-of-signal vs. Background			Interference vs. Background		
**Name**	**Relation**	**AUC**	**Name**	**Relation**	**AUC**

*n*	LOS > Bg	0.93	*n*	I > Bg	0.88
*k*_3_	LOS > Bg	0.86	*K*	I > Bg	0.77
*K*	LOS > Bg	0.84	*s*_1_	I > Bg	0.76
*t*_1_	LOS > Bg	0.83	*s*_2_	I > Bg	0.75
*s*_2_	LOS > Bg	0.70	*s*_3_	I < Bg	0.71
*k*_5_	LOS > Bg	0.67	*k*_2_	I > Bg	0.67
*t*_4_	LOS > Bg	0.66	*d_W_*	I > Bg	0.66
*k*_1_	LOS > Bg	0.65	*k*_5_	I > Bg	0.66
*t*_3_	LOS < Bg	0.64	*t*_3_	I > Bg	0.66
*s*_3_	LOS < Bg	0.63	*t*_4_	I > Bg	0.61
*s*_4_	LOS < Bg	0.61	*k_W_*	I < Bg	0.60
*d_w_*	LOS > Bg	0.61	*k*_7_	I < Bg	0.58
*k*_7_	LOS < Bg	0.58	*k*_4_	I < Bg	0.58
*k_W_*	LOS < Bg	0.57	*s*_4_	I < Bg	0.57
*k*_2_	LOS > Bg	0.57	*k*_1_	I < Bg	0.57
*k*_4_	LOS < Bg	0.56	*k*_3_	I > Bg	0.56
*t*_2_	LOS > Bg	0.56	*k*_6_	I > Bg	0.56
			
*k*_6_	LOS > Bg	0.54	*t*_2_	I > Bg	0.54
*s*_1_	LOS > Bg	0.51	*t*_1_	I < Bg	0.53

The results from the analysis step, comparing parameter value distributions of the two models with the background distribution. Parameters are sorted by area under ROC curves (AUC). The parameters under the line have a p-value greater than 0.05 and are not statistically significant, where p-values are calculated with a *Wilcoxon rank-sum test*. See also Fig. 5.

To highlight features of each model we studied the parameters that differed the most in more detail to find out how they relate to biological mechanisms. We noted that the Hill parameters (*K *and *n*) were among the top candidates for both hypotheses (Table 3). These parameters tune the regulation of WUS by the combined pathway (Methods). The validation step did introduce large constraints on the parameters (Figure 3), and it is likely that fitting the parameters to the double mutant experiments led to a tighter regulation of the Hill parameters. To confirm this, we did an AUC parameter comparison between data after the optimization step and data after the validation step, wherein both Hill parameters appeared among the top three parameters (data not shown). We also did an AUC comparison between the two models' data sets, and in this case the Hill parameters did not show up at the top of the list (data not shown). As a consequence of these results we did not look further into these two parameters, but focused on the receptor ligand dynamics.

We chose, somewhat *ad hoc*, to make a cut in AUC at 0.75 for this analysis. The comparison between the loss-of-signal hypothesis and the background emphasized two parameters. the strength of the CLV1/CLV3 signal into the cell (*k*_3_) and the CLV1 turnover rate (*t*_1_) (Figure 5B-C). The comparison between the interference hypothesis and background also highlighted two parameters, which in this case were the production rates for the two receptors (*s*_1_, *s*_2_) (Figure 5D-E).

In conclusion we have shown that parameter calibration of the different implementations of the *clv1-1 *mutants imposes non-expected constraints on the parameters of the two models, and in the further analysis we found four parameters that were the most discriminating between the models. These four parameters will be used in the proceeding analysis to evaluate their consequences from a biological perspective.

### The WUS-repressing signal is dependent on both receptor pathways with a slightly stronger CLV1-dependence

The parameter that differed the most in the comparison between the loss-of-signal and background distributions was *k*_3 _(Table 3), wherein it was larger for the loss-of-signal model. This is not surprising since *k*_3 _sets the strength of the signal from the bound CLV1 receptor and the loss-of-signal mutant needs to be able to reduce this signaling strength for the *clv1-1 *mutant.

The selection of *k*_3 _may also be an indication that it is important for the loss-of-signal model that the CLV1/CLV3 pathway is stronger than the CRN/CLV3 pathway. In our implementation the strength of the former is given by *k*_3 _times the concentration of bound CLV1 receptors, and the latter is given by *k*_6 _times the concentration of bound CRN receptors. We observed that the CLV1 pathway indeed was slightly stronger for both models (Figure 5FH) and this effect was somewhat stronger for the loss-of-signal model. However, the asymmetry between the two pathways was fairly small and it is obvious that the WUS-repressing signal is dependent on both pathways. By only considering the wild type network, one might assume that using double pathways would mean that the strengths of the two could be freely tuned. if the strength of one of the pathways is increased, the increase could be compensated by decreasing the other to obtain the same combined signal. This was however not what we observed in our analysis. In contrast, when also taking the mutants into account it is important to divide the signal evenly between the two pathways, but with a slightly stronger CLV1 pathway signal (Figure 5F-H). This is true for both models, as was also suggested by the mutant experiments since the *clv1 *phenotypes are slightly stronger than the *crn *phenotypes (Table 1).

### Degradation or internalization of the bound receptor is implicated by the loss-of-signal clv1-1 hypothesis

The second parameter highlighted for the loss-of-signal model, from the comparison with the background distribution, was *t*_1 _(Table 3). The *t*_1 _parameter sets the turnover rate of the CLV1 receptor, including the degradation of the bound receptor (Methods). The loss-of-signal model typically had a larger value of this parameter. Interestingly, this was in accordance with our finding that the loss-of-signal model without degradation of the bound CLV1 receptor could not cohere with all mutant data simultaneously (data not shown). Together these results predict that for the loss-of-signal model to work the bound CLV1 receptor needs to be removed from the membrane, possibly via internalization.

Receptor trafficking has been suggested to be important for several cell-signaling pathways [28] and endosomal functions play major role in plants [29]. Although there has been no experimental evidence for CLV1 internalization, other receptors such as FSL2 and BRI have been shown to be internalized [29]. Recent GFP-data of CLV1 indicates that CLV1 is located mainly in the plasma membrane but can also be found in internal cell compartments [10]. The model suggests that there should be a non-negligible rate of internalization of the bound CLV1 receptor.

### The interference hypothesis leads to large quantities of receptors

**Figure 6:**
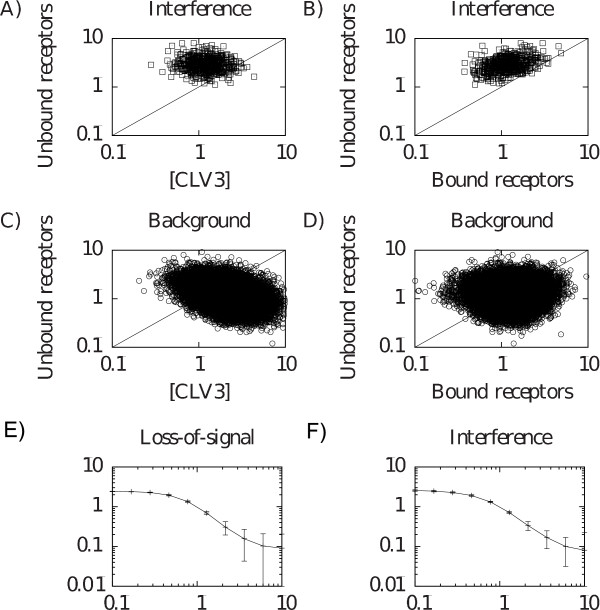
**Receptor concentrations from simulations and CLV3 perturbation simulations**. A-D) Study of the amount of free receptors compared with the amounts of bound receptors and free CLV3. A and C) Concentrations of free CLV3 compared with amounts of free receptors in simulations of wild type with the interference model (A) and the background distribution (C). B and D) Amounts of bound receptors compared with amounts of free receptors in simulations of wild type with the interference model (C) and the background distribution (D). E-F) Study of CLV3 over- and underexpression. The parameters *s*_3 _and *k_W _*are multiplied by a factor (presented as the abscissa). The perturbed WUS expression is then normalized with the unperturbed WUS expression (presented as the ordinate). Presented data is average and standard deviation of normalized equilibrium WUS expressions in simulations of wild type with the loss-of-signal model (E) and the interference model (F).

From the comparison between the interference model and the background distribution it was seen that the parameters for receptor production, *s*_1 _and *s*_2_, are large in the interference model (Table 3). This suggests that the interference model requires large quantities of receptors to reproduce the differences in phenotype of the *clv1-1 *non-null mutant and the *clv1-11 *null mutant. An explanation could be that there must be enough receptors available for the interference mechanism to be efficient. When comparing the amount of free receptors with the amount of free CLV3 in wild type simulations, we observed that there was an abundance of receptors (Figure 6A). It can be noted that large quantities of both receptors are present (data not shown). As a comparison, the background distribution data did not show any bias towards an abundance of free receptors (Figure 6C).

In addition, we compared the amount of bound versus free receptors (Figures 6B and 6D), and only the interference model data displayed a bias towards more free than bound receptors in the wild type simulations. From a biological point of view, the strategy of having large amounts of free receptors might not be advantageous given the metabolic cost associated with the production of receptors. However, the optimization algorithm does not take metabolic costs into consideration and it might be that, if it had, the result could be different. The addition of metabolic cost would be an interesting improvement of the optimization algorithm that could be tested.

At this point, we have examined all parameters highlighted by the AUC value, but our threshold value for choosing parameters to scrutinize was somewhat *ad hoc*. Hence it could be of interest to briefly investigate the parameters that follow in the sorted list (Table 3). The CLV3 production rate (*s*_3_) is lower and the unbinding rate of the CLV1/CLV3 complex (*k*_2_) is higher for the interference model compared to the background distribution, acting in directions of having low CLV3 and much unbound receptor, strengthening the conclusions in this section. For the loss-of-function model the CRN production rate (*s*_2_) and the unbinding rate of the CRN/CLV3 complex (*k*_5_) are high, acting in the direction of having much unbound CRN, which we could see in the simulated data (data not shown). Unbound CRN acts in favor of CLV3 sequestration in the *clv1-1 *mutant, strengthening our sequestration discussion above.

In conclusion, our model analysis pinpointed the receptor abundance as important for both receptors in the interference model and also for the CRN receptor in the loss-of-function model. It would be interesting to see how these predictions relate to experimental data, especially the somewhat counterintuitive prediction that there is a large pool of inactivated receptors.

### Additional perturbations

Although the CLV3 feedback is simplified in the model (Methods), it is of interest to analyze the model behavior for different CLV3 perturbations since these have been extensively studied in experiments. Loss-of-function *clv3 *mutants show an increase in WUS expression, and gain-of-function mutants repress WUS [4-6]. Simulated perturbations where CLV3 production was changed an order of magnitude up and down are presented in Figure 6E. The models correctly predicted an increase of WUS as the CLV3 was lowered and a decrease when CLV3 was increased. However, it has been shown in more detailed CLV3 perturbation experiments that it is possible to change CLV3 expression strength over a large range without any apparent phenotypical effects [30]. This was not captured in our CLV3 perturbation simulations (Figure 6E-F) and hence the model only qualitatively predicted the perturbations. This did not come as a surprise since we have focused our investigations on receptor mutants and we used a direct linear WUS-dependent activation of CLV3. In reality CLV3 is expressed in different cells compared to WUS and the (still unknown) regulation is possibly nonlinear. To introduce a spatial model would however require additional assumptions and hypotheses (Methods).

We also did a sensitivity analysis of individual parameters and both models were robust to changes in most parameters (Table 2, Methods), an important feature given that the stem cell regulation needs to be robust to environmental changes and stable over long periods of time.

## Conclusions

The stem cell regulation network in the plant shoot apical meristem is a well-studied system given its necessity for aboveground plant development. The large amount of mutant phenotype data available together with several gaps in knowledge of network details call for computational modeling as a tool for understanding the complex regulation at a systems level.

We have developed a model for the stem cell regulation network based on the negative feedback loop between CLV3 and WUS and focused on CLV3-receptor dynamics and mutants. The model takes into account a negative regulation of WUS via two different - although merging - receptor pathways, CLV1 and CRN, as well as a WUS-induced CLV3 production. Starting from the question how the *clv1-11 *null mutant can be weaker in phenotype compared to the non-null *clv1-1 *mutant, we scrutinized two models implementing different hypothesized mechanisms for the *clv1-1 *mutant.

In the first tested mechanism for *clv1-1*, the mutated *CLV1 *product interfered with the CRN pathway, as previously suggested [30]. Due to the interference, the CRN signal is decreased in the *clv1-1 *mutant, as compared to the null mutant. Since both pathways are decreased the phenotype becomes stronger. We could also conclude that this hypothesis led to the prediction that there is an abundance of receptors in relation to CLV3.

In the second model the *clv1-1 *mutant was implemented as a loss-of-signal mutant, i.e. the receptor still binds CLV3 but no signal is propagated. We showed that the CRN signal is decreased in the *clv1-1 *mutant compared to the null mutant due to sequestration of CLV3 by the continued binding to the CLV1-receptor. For this model to work it is necessary that the bound CLV1 receptor is internalized or degraded.

The adopted approach for extracting parameter ensembles and using statistical methods when comparing different hypotheses is generally applicable for systems biology modeling and provides an objective approach for dealing with unknown parameters. A future improvement could be to adopt machinelearning techniques of cross-validation by selecting different mutants to be included in the optimization and validation sets and then merge into a single parameter ensemble.

Our model represents a simplification of the system, most notably by disregarding spatial factors and not including all possible receptors and additional mechanisms known to be important for WUS regulation. However, these simplifications allowed us to investigate multiple receptors with an almost complete set of receptor mutants. We were not able to discard any of the two *clv1-1 *hypotheses, but still we were able to deliver experimentally verifiable predictions for both hypotheses, also on general properties of the stem cell regulating network that are indirect consequences of the hypotheses that would have been difficult to predict by intuition alone. The loss-of-signal mechanism has the advantage that it is more parsimonious since it only affects the signaling capacity, which fits well with the allele's known mutation in the kinase domain. The interference mechanism, on the other hand, is less constrained and seems to have an easier task in producing the mutant results. In the end it is experiments that should resolve the issue and we hope that this investigation can serve as an inspiration both for new experiments and for modelers to include multiple receptors in models of plant stem cell regulation.

## Methods

### Models

In our models CLV3 binds to the receptors CLV1 and CRN and thereby activates them. Activated CLV1 and CRN contribute to a signal X, which inhibits expression of WUS. WUS promotes production of CLV3 and therefore enables for a selfregulating system. CLV3, the receptors CLV1 and CRN, and the signal X all have a basal level of production, while WUS has basal production that can be repressed by the X signal. All molecules have a concentration-dependent degradation rate. See Figure 1A for a schematic drawing of the wild type model. We use mass action kinetics to get a mathematical representation of the model in the form of a system of ordinary differential equations. For the wild type case the equations are

(1) d[CLV1]dt=t1(s1−[CLV1])−k1[CLV1]⋅[CLV3] +k2[CLV1/CLV3]

(2) d[CRN]dt=t2(s2−[CRN])−k4[CRN]⋅[CLV3] +k5[CRN/CLV3]

(3) d[CLV3]dt=t3(s3−[CLV3])+kW[WUS] −k1[CLV1]⋅ [CLV3] +k2[CLV1/CLV3] −k4[CRN]⋅ [CLV3] +k5[CRN/CLV3]

(4) d[CLV1/CLV3]dt=k1[CLV1]⋅[CLV3] −k2[CLV1/CLV3] −t1[CLV1/CLV3]

(5) d[CRN/CLV3]dt=k4[CRN]⋅[CLV3] −k5[CRN/CLV3] −t2[CRN/CLV3]

(6) d[X]dt=t4(s4−[X])+k3[CLV1/CLV3] +k6[CRN/CLV3]

(7) $$\frac{d[\text{WUS}]}{dt}=k_{7}\frac{K^{n}}{K^{n}+{\left[\text{X}\right]}^{n}}-d_{W}[\text{WUS}],$$

where [CLV1] and [CRN] are concentrations of unbound receptors, [CLV3] is the concentration of the CLV3 peptide, [CLV1/CLV3] and [CRN/CLV3] are concentrations of bound receptors, [X] is the concentration of the signal X, and [WUS] is the concentration of the transcription factor WUS.

The parameters *k*_1_, *k*_2_, *k*_4_, and *k*_5 _are reaction constants for binding of CLV3 to CLV1 and CRN. The parameters *k*_3 _and *k*_6 _set the strengths of the signals from the activated receptors to the signal X. Production and degradation of CLV1, CRN, CLV3, and X are determined by the parameters *t*_1_, *s*_1_, *t*_2_, *s*_2_, *t*_3_, *s*_3_, *t*_4_, and *s*_4_, while *d_w _*sets degradation of WUS. Production of WUS, downregulated by X, is controlled by parameters *k*_7_, *K*, and *n*. Note that we do not have experimental estimates of parameter values and it is mainly the relation between parameters that is of importance; hence we refrain from specifying unit values on our parameters.

Our model is to be seen as a simplification of the SAM. The SAM is divided into several spatial regions each characterized by different gene expression patterns. Cells in the SAM also divide with time and move between different regions. We do not address the positioning associated with the different expression domains. While WUS, CLV1, and CRN are expressed in the same cells, CLV3 is not [2,3]. We include a simplified CLV3 feedback via a direct connection from WUS and a direct binding of CLV3 to the receptors. The rationale for this simplification is that the way the spatial signals are propagated is not fully understood and would require adding multiple additional hypotheses (see e.g. [21-23]). Our implementation still covers the main feedback interactions needed to investigate the behavior of the different receptor mutants that are the main target of this work. The internal part of the signaling pathway is in our model described by a single signal X. Although the POL/PLL1 has been shown to be important for mediating the signal [14], the details of the architecture is unknown and we point this out by instead representing the signal with X.

The state of the CLV signaling network is in our model measured via WUS expression levels. The expression of WUS and CLV3 is altered in the SAM when CLV signaling is perturbed, both by expanding/decreasing expression regions and by increasing/decreasing levels [18]. Our WUS measure serves as a simplification of this.

#### Mutants

We are interested in several receptor mutants and we implement them in different ways. The *clv1-11 *and *crn-1 clv2-1 *mutants are both modeled by removing all presence of CLV1 ([CLV1] = [CLV1/CLV3] = 0) and CRN ([CRN] = [CRN/CLV3] = 0) respectively. The *crn-1 *mutant is modeled by setting *k*_6 _= 0. The remaining mutant of interest - *clv1-1 *- is treated differently in two models.

In the loss-of-signal model the parameter *k*_3 _is replaced by another parameter *k*_3_,_weak _for the *clv1-1 *mutant (Figure 1B).

In the interference model an extra mechanism is introduced (Figure 1C). For the *clv1-1 *mutant, the receptors CLV1 and CRN can form a complex and when they do the complex is discarded. Mathematically an extra term is added to the time derivative of CLV1 and CRN,

(8) $$\frac{d[\text{CLV}1]}{dt}=\ldots -k_{8}[\text{CLV}1]\cdot [\text{CRN}]$$

(9) $$\frac{d[\text{CRN}]}{dt}=\ldots -k_{8}[\text{CLV}1]\cdot [\text{CRN}].$$

### Computational Procedures

Our approach can be divided into three steps; an optimization step, a validation step, and an analysis step. In the optimization step we use an optimization algorithm to find parameter (value) sets for which the models can reproduce the wild type and the four loss-of-function mutants. In the validation step we choose the subset of these parameter sets for which the models also explain the two double mutants. In this way we reduce the problem of over- fitting to data, which usually hampers the predictive power of the models.

After the validation step a large number of possible parameter sets remain. Instead of the more usual approach of just choosing the parameter set for which the model best matches experimental data, we keep all parameters and look at the ensembleoutput of the model. In this way we get semi-global robust predictions of the model behavior. However, this leaves us with lots of possible parameter sets that require further analysis. In the analysis step of our computational approach we deploy a number of computational and statistical tools to analyze the ensemble of obtained parameter sets. In this way we are trying to further analyze the behavior of the models to find significant differences.

### Optimization

A Simulated annealing algorithm was used to fit parameters to experimental data [31]. The algorithm is divided into four steps.

1. An initial set of parameters, *p*_initial_, is randomly chosen from a uniform distribution.

2. A proposed new set of parameters *p*_new _is chosen by randomly picking a parameter and changing its value by multiplying or dividing (with equal probability) with a factor 1.01. An associated energy is calculated by a parameterdependent energy (objective) function *E *= *E*(*p*) (see below).

3. The new parameter set, *p*_new_, is kept with a probability $P=\min(1,e^{-\beta (E(p_{new})-E(p_{old}))})$, where *β *is a positive parameter inversely proportional to a virtual temperature.

4. Step 2-3 is repeated while *β *is being increased every thousand iterations by a factor 1.1, starting from 1 until it ends at 10,000.

At each iteration, the energy associated with the current parameter set is compared with the overall best performing parameter set, i.e. the parameter set with the lowest corresponding energy. When *β *reaches its maximum value the algorithm stops and the best performing parameter set is used as the solution to the optimization problem.

Each optimization gives a proposed parameter set. After each optimization the parameter set is tested with the model against the experimental data. If the model is able to reproduce the experimental data within the errors supplied the parameter set is kept, otherwise it is discarded.

#### Energy function

To compare phenotypic strength between models and experiments, we use WUS levels as a measure in the models and compare with carpel numbers from experiments. Carpel numbers have been used extensively in the literature as a measure of the phenotypic strength of perturbations to the CLV signaling network (see e.g. [9,19], and [15] for an example where both RT-PCR measurements of WUS and carpel numbers are reported).

To calculate the energy for a given parameter set we first calculate the equilibrium of WUS concentration, [WUS]*, for the wild type experiment, and for the *crn-1*, *clv1-11*, *crn-1 clv2-1*, and *clv1-1 *mutant experiments. The WUS levels for the mutant experiments are normalized with the wild type WUS level. The energy function is defined as

(10) $$E=\sum_{i}{\left({\left[{\text{WUS}}_{i}\right]}^{*}-D_{i}\right)}^{2},$$

where [*WUS_i_*]* is the normalized equilibrium WUS expression for experiment *i*, *D_i _*is the expected value from experiment, and the summation is over all mutant experiments. The experimental values that we have used to find parameters are presented in Table 1[9].

### Validation

To reduce overfitting we leave two double mutant experiments out of the optimization step and instead use them for a validation step. Simulations of two double mutants *crn-1 clv1-11 *and *crn-1 clv1-1 *for the two models are compared with experimental data to find parameters that can be used to reproduce the behavior of both the single and double mutant experiments. In the validation step we use a larger threshold for validating simulations compared to what was used in the optimization step (Table 1).

### Numerical solutions

We are interested in fixed point solutions to the system, which are obtained by solving the system of equations when all time derivatives are equal to zero. At equilibrium the fixed point concentrations [X]*, [CLV1/CLV3]*, and [CRN/CLV3]* are equal to

(11) $${\left[\text{X}\right]}^{*}=s_{4}+\frac{k_{3}{\left[\text{CLV}1/\text{CLV}3\right]}^{*}+k_{6}{\left[\text{CRN}/\text{CLV}3\right]}^{*}}{t_{4}},$$

(12) $${\left[\text{CLV}1/\text{CLV}3\right]}^{*}=\frac{k_{1}}{k_{2}+t_{1}}{\left[\text{CLV1}\right]}^{*}\cdot {\left[\text{CLV}3\right]}^{*},$$

(13) $${\left[\text{CRN}/\text{CLV}3\right]}^{*}=\frac{k_{4}}{k_{5}+t_{2}}{\left[\text{CRN}\right]}^{*}\cdot {\left[\text{CLV}3\right]}^{*}.$$

The three fixed point concentrations [CLV1]*, [CRN]*, and [CLV3]* are given by the solution to the system of equations

(14) t1(s1−[CLV1]∗)−b1[CLV1]∗⋅[CLV3]∗−k8[CLV1]∗⋅[CRN]∗=0,

(15) t2(s2−[CRN]∗)−b2[CRN]∗⋅[CLV3]∗−k8[CLV1]∗⋅[CRN]∗=0,

(16) t3(s3−[CLV3]∗)−b1[CLV1]∗⋅[CLV3]∗−b2[CRN]∗·[CLV3]∗+kW[WUS]∗=0,

with *k*_8 _≠ 0 for the *clv1-1 *mutant in the interference model and *k*_8 _= 0 otherwise, and where

(17) $$b_{1}=\frac{t_{1}k_{1}}{k_{2}+t_{1}}\;\text{and}\;b_{2}=\frac{t_{2}k_{4}}{k_{5}+t_{2}}.$$

The equilibrium expression of WUS, [WUS]*, is the solution to

(18) $$k_{7}\frac{K^{n}}{K^{n}+{\left[\text{X}\right]}^{*n}}-d_{W}{\left[WUS\right]}^{*}=0,$$

To numerically find the equilibrium concentrations we first consider Eq. 18 as a function *f *of WUS expression

(19) $$f([WUS])=k_{7}\frac{K^{n}}{K^{n}+{\text{X}}^{*}{\left([\text{WUS}]\right)}^{n}}-d_{W}[\text{WUS}],$$

where X* = X*([WUS]) is a function of WUS given by Eqs. 11-16. The equation *f*([WUS]) = 0 is solved numerically by the bisection method [31]. As an intermediate step we solve the system of equations, Eqs. 14-16, with Newton's method [31]. We define equilibrium as follows; the Newton's method iterates until |**e**| < 0.001, where

(20) $$e=\left(\frac{d[\text{CLV}1]}{dt},\frac{d[\text{CRN}]}{dt},\frac{d[\text{CLV}3]}{dt}\right),$$

and the bisection method iterates until |*f*([WUS])| < 0.0001.

### Sensitivity analysis

The models' robustness to parameter perturbations were tested by a sensitivity analysis [32]. If *M *is a quantity of the system and *p *is a parameter, the sensitivity *S_p _*is defined as

(21) $$S_{p}=\frac{\partial M}{\partial p}\frac{p}{M}.$$

The absolute value of *S_p _*serves as a measurement of how sensitive *M *is to perturbations in parameter *p*. A greater value corresponds to a greater sensitivity. The sign of *S_p _*tells if the response is positive or negative in respect to a positive change of the parameter. We use the equilibrium WUS concentration for the wild type network for our measurable quantity during our sensitivity analysis (Table 2). Both models were robust to changes in most parameters. The only parameters that had a sensitivity significantly above one were *K *and *t*_4_, both parts of the pathway between the signal and the WUS expression. This part of the model is a crude approximation of the internal pathway architecture. The internal signaling pathway was also highlighted when comparing differences in parameters (cf. *n *and *K *in Table 3), which indicates the importance to further investigate this part of the network, both in experiments and by modeling.

### Receiver operating chacteristic curves

A receiver operating characteristic (ROC) curve measures the overlap of the distributions of two data sets A and B. The area under the ROC-curves (AUC) quantifies differences between A and B independent of the number of parameter sets within the distributions [27]. By calculating the AUC we get a value between 0 and 1, where 0 means that the values of data set A are all greater than those of data set B, 0.5 means that the two sets come from the same distribution, and 1 means that all values of data set B are greater than those of data set A.

## Availability

The optimization algorithm, the numerical solver, and the statistical tools are in house implementations and are publicly available upon request. Operating system(s). Platform independent; Programming language. C++; Licence. no licence needed.

Parameter data can be found at http://www.thep.lu.se/~henrik/clvCrn/.

## Authors' contributions

PS and PM implemented and applied simulation and analysis tools. All authors designed experiments, analyzed data, and wrote the paper.
